# Unraveling Shulman's syndrome: A rare case of eosinophilic fasciitis in a pediatric patient with fascial abnormalities on MRI

**DOI:** 10.1016/j.radcr.2024.10.123

**Published:** 2025-01-10

**Authors:** Hadj Hsain Ihssan, Ezzaky Sara, Maslouhi Kaoutar, Lahlou Chailmae, Marrakchi Salma, Allali Nazik, Chat Latifa, El Haddah Siham

**Affiliations:** Departement of radiology HER, University Mohammed V Rabat, Rabat, Morocco

**Keywords:** Shulman's Syndrome, Eosinophilic Fasciitis, MRI

## Abstract

We report the case of a 15-year-old girl who presented with a 2-month history of severe fatigue and rapidly worsening myalgia. Biological tests revealed hypereosinophilia and an inflammatory syndrome. MRI showed increased signal intensity in the superficial and deep aponeurotic layers on T2-weighted images, with moderate fascia enhancement after contrast administration. A muscle biopsy of the arm was performed, revealing an accumulation of eosinophils in the muscle aponeuroses. Eosinophilic fasciitis (EF) is a rare connective tissue disorder characterized by inflammation of the fascia, leading to skin thickening and limb pain. While it predominantly affects adults, pediatric cases are rare, making diagnosis challenging due to its overlap with other fibrotic diseases.

## Introduction

Eosinophilic Fasciitis (EF) is a rare connective tissue disorder with an unknown etiology and poorly understood pathophysiology. It was first described in June 1974 at the Sixth Pan-American Rheumatology Congress in Toronto by Lawrence E. Shulman [1]. Shulman reported cases of 2 adult men with a novel syndrome characterized by diffuse subcutaneous induration that appeared a few days after unusual physical exertion, blood eosinophilia, elevated erythrocyte sedimentation rate (ESR), and hypergammaglobulinemia. Skin and muscle biopsy showed thickening of the muscle fascia. Shulman named this syndrome “fasciitis with eosinophilia (FE)”. One year later, in 1975, Rodnan et al. [2] described a similar clinical picture in 7 other patients. Unlike Shulman, they found eosinophils in the fascia infiltrate and named this syndrome “eosinophilic fasciitis”. Since then, hundreds of publications have been dedicated to Shulman's syndrome.

For the diagnosis and monitoring of this condition, MRI is the preferred modality as it shows typical signal abnormalities and thickening of deep fasciae [3]. Confirmation of the diagnosis is through deep skin biopsy, revealing chronic inflammatory infiltration affecting the deep fascia with lymphocytes, histiocytes, and occasionally eosinophils.

In this report, we aim to present the clinical characteristics and MRI findings for the diagnosis of Shulman's syndrome, along with a brief literature review.

## Case report

**Fig. 1:**
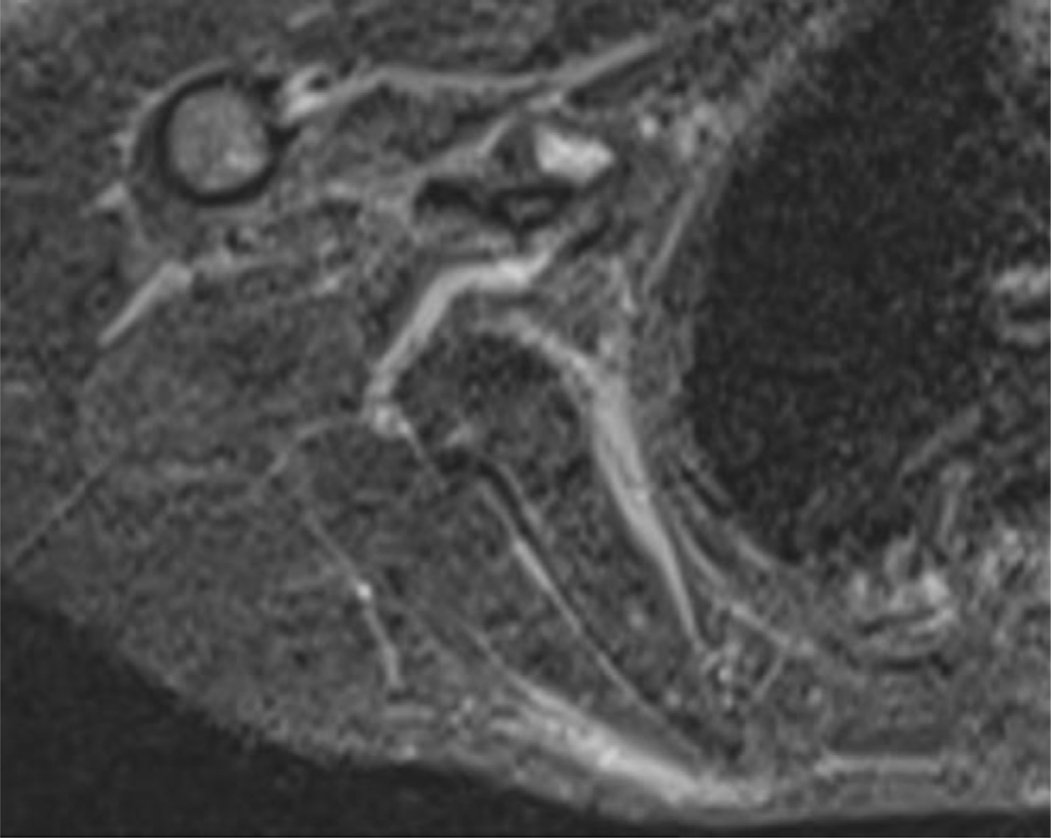
Axial section of T2 sequence with suppression of arm fat signal showing hyper signal of sub-scapular muscle fascia.

**Fig. 2:**
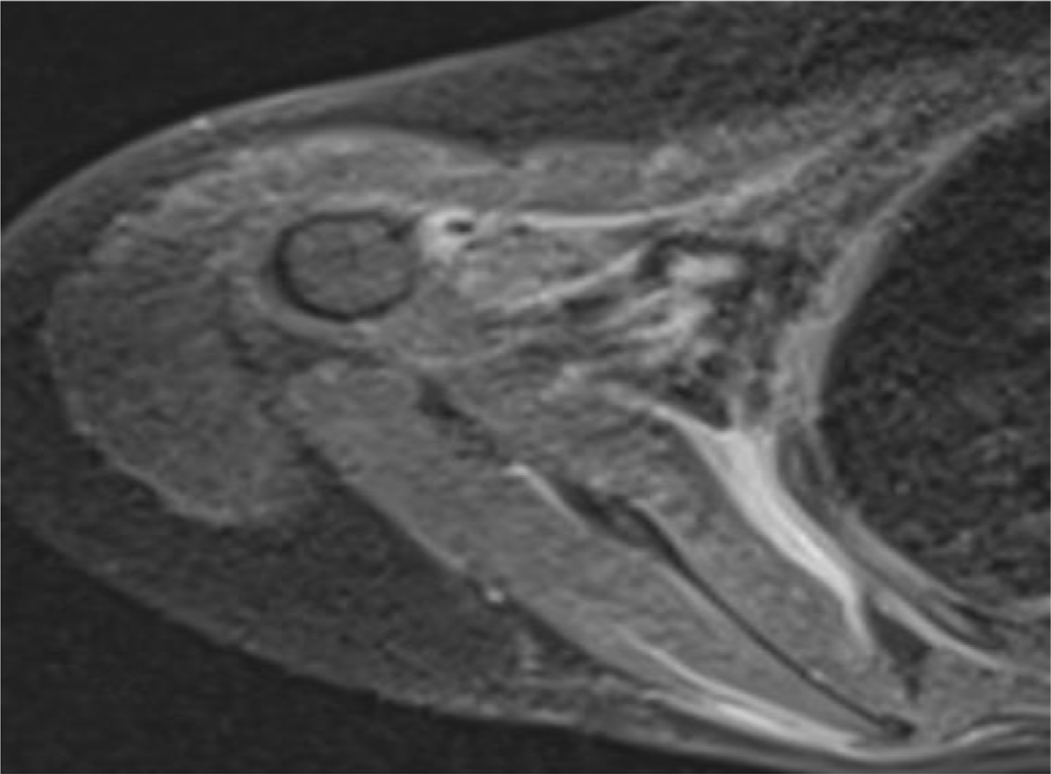
Axial section showing intense enhancement of sub-scapular muscle fascia after GADO injection on T1 sequence with suppression of fat signal.

**Fig. 3:**
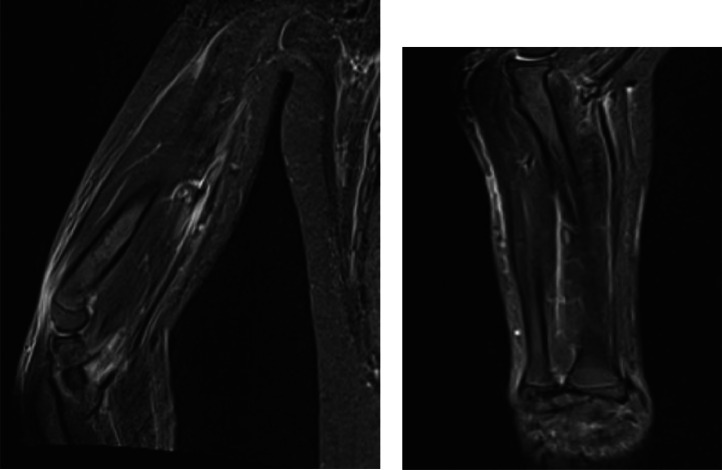
Coronal sections of the arm and forearm showing diffuse hypersignal of muscle fascia.

**Fig. 4:**
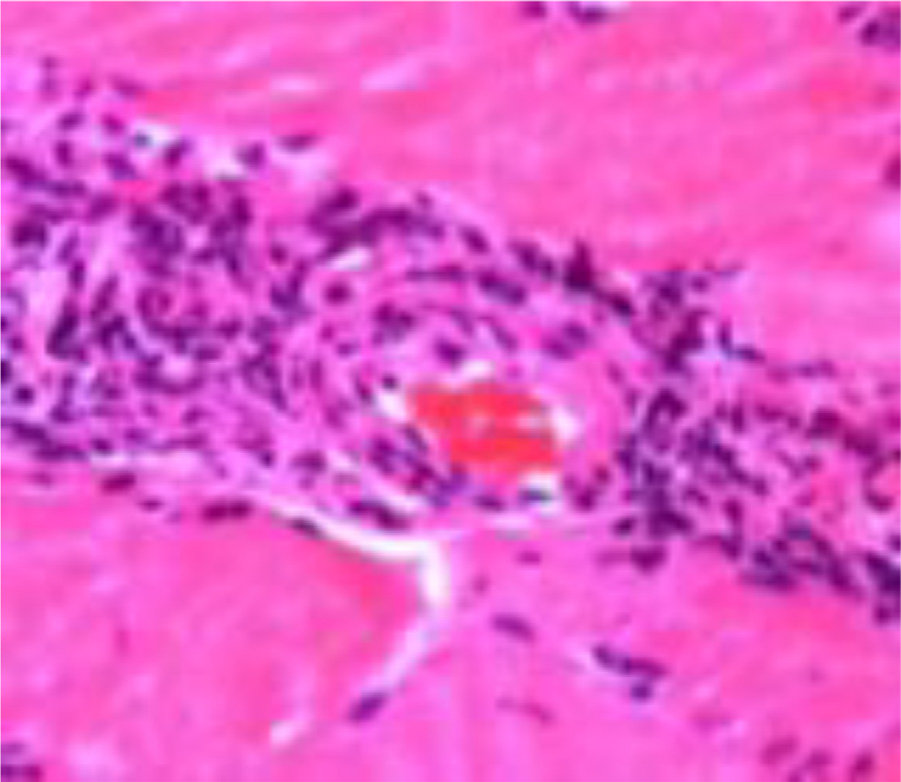
Cutaneous-fascial-muscular biopsy of a patient with FE: hematoxylin-eosin staining shows dense, diffuse, perivascular inflammatory infiltrates in the fascia, composed mainly of lymphocytes, but also of eosinophils.

A 15-year-old girl presented with a 2-month history of severe fatigue, rapidly worsening myalgia, and painful swelling of the forearms. Physical examination revealed skin tightening of the upper limbs, with both elbows fixed at a 90-degree dorsiflexion angle, without signs of arthritis in the joints. Neurological examination of the patient was normal. Laboratory tests showed a white blood cell count of 12,5000/µL, ([normal: 4500–11,000/µL], with eosinophils 1500/µL (normal: 50-500/µL). ESR was 79 mm/h (normal: <20 mm/h), and C-reactive protein (CRP) level was 80 mg/dl (normal: <5 mg/L). Liver, renal, and thyroid function tests were normal. Uric acid levels were within the normal range. Rheumatoid factor and antinuclear antibodies (ANA) tests were negative. Protein electrophoresis revealed polyclonal hypergammaglobulinemia. MRI showed increased signal intensity in superficial and deep fascia layers on T2-weighted images (Figs. 1 and 3) with moderate fascia enhancement after contrast administration (Fig. 2), a muscle biopsy of the arm was performed showing an accumulation of eosinophil in the muscle aponeuroses (Fig 4). our patient was put on corticosteroid boluses as well as immunosupressants such as metotrexate, after 1 month of treatment there was a clear improvement in clinical signs as well as biology results showing a decrease in eosiniphilia, CRP and ESR.

## Discussion

Eosinophilic fasciitis (FE) is a rare disease of the connective tissue whose etiology and physiopathology remains unknown, to be considered as a variant of scleroderma, without Raynaud phenomenon nor telangiectasia or extra-cutaneous manifestation [4], The median age for the onset of the disease is approximately 40-50 years, with no gender predilection, but likely affecting men earlier [4,5].

The physiopathology of FE remains largely obscure. However, an autoimmune mechanism is suspected due to the presence of immune deposits (IgG,IgM, C3, C4) in the fascias, circulating immune complexes, and the possible but unconstant existence of antinuclear antibodies and rheumatoid factors [6]. The immune cells accumulated in the fascia and dermis (lymphocytes, histiocyte, plasmocyte and eosinophile). Lymphocytes, by the production of cytokines, and eosinophils by their degranulation and release of beta TGF, are responsible for increased proliferation of fibroblasts, and their synthesis of more collagen and extracellular matrix proteins [7].

On the other hand, the pathogenesis seems to be multifactorial: intense physical effort or muscle trauma before the onset of symptoms is often one of the most important factors in patogenesis [8,9], Some cases have been after taking the following medicines (simvastatin, atorvastatine, phenytoin, ramipril, subcutaneous heparin, fosinopril, alpha-methyldopa, antituberculosis therapy and, more recently, nataluzimab) or after chemical exposure [10,11]. In other cases, the condition has been associated with a bacterial infection (borreliosis, mycoplasma) [11].

Clinically,The edema often appears abruptly and early with or without the sign of the bite [12], which can be painful and inflammatory,It is often associated with itching and urticary rash.

The edema is then replaced by a sclerodermiform subcutaneous hardening. The skin thickens and compresses, it is no longer possible to move it on the underlying planes or to pinch it, due to this hardening, the patient experiences a feeling of stretching, internal tension or carapace [13].

Irregular adhesions to deep planes create a folding effect ("orange skin" or “sand dunes”), especially visible on the front side of the arms and thighs.Aponevrotic retractions cause the veins of the upper limbs, instead of pumping outward, to run in the form of depression (the sign of the valley).

Topographically, topographically, the skin damage is generally bilateral and symmetrical and centripetal, although unilateral forms have been [10], It affects the hands in 30%-54% of cases and the lower limbs in more than 70% and the forearms and arms in 85%-90% of cases as in our patient.[8.10], other localizations have been described, including the cervical region (6%-19%), the abdomen (23%-44%) and the thorax.and the back (6%-38%), more exceptionally the face [8,10].

In order to identify the peculiarities of Shulman syndrome in paediatrics, Farrington et al. [14] studied the manifestations of the Syndrome in 21 children. They concluded that this syndrome is more common in girls and that it affects the hands more often than the joints, which is the case with our patient. Seventy-seven percent of cases progress to residual skin fibrosis, especially in children under the age of 7 and those with a widespread form of the disease. None progresses to systemic scleroderma.

Skin manifestations are associated with myalgia in 86% of patients and joint lesions with inflammatory arthralgia in almost 40% of patients [8].

Eosinophilic fasciitis has been frequently associated with hematological disorders (in 10% of patients), such as aplastic anemia, hemolytic anaemia, thrombocytopenia, leukemia, lymphoma, and other myeloproliferative disorder [15].

Shulman syndrome associated with hemopathies can be fatal or regress with treatment [15]. In contrast, cases of Shulman syndrome in the paraneoplastic context, associated with solid cancers, such as breast cancer [16] or gastric adenocarcinoma [17], have been in which the treatment of the tumor resulted in remission of the syndrome.

On the biological level, laboratory tests can reveal peripheral eosinophilia in 60%-90% of patients [[9], [10], [11]]. However, it is not essential for the diagnosis of FE and its number is not associated with the prognosis of the disease [5,10]. Inflammatory syndrome is frequently with high C-reactive protein (50%-64%), an increase in erythrocytes sedimentation rate (30%-60%) and generally polyclonal hypergammaglobulinemia (35%-60%), as in our case [9,10]. An increase in aldolase was also in 31% of patients [10]. Increase in serum creatinine phosphokinase is rare (4%-10%) and may reflect moderate muscle loss during FE [9,10]. The search for antinuclear antibodies is positive in 15%-20% of cases, but no anti-DNA antibody or ANCA [9,10]. MRI is now considered the best imaging tool for the morphological diagnosis of FE. Typically, MRI shows a thickening of deep fascias on T1 weighted sequences that appears with an increased signal intensity on fluid sensitive sequences (fat-weighted T2 weighted Sequences), and a marked increase after gadolinium injection in the acute phase of the disease [9,10,18,19], less frequently MRI signal abnormalities can be observed in the muscle and hypodermal tissue adjacent to the fascia [19], the same aspect was found in our patient. MRI may be normal when performed very early or after the start of corticosteroid therapy [10]. MRI is also useful to guide the surgeon to the optimal location of the biopsy, thereby reducing the risk of sampling errors and false negative results [18]. MRI is also used to monitor the response to corticosteroid therapy [10,17,20].

Deep skin biopsy (skin and muscle) is the gold standard for the diagnosis of FE [21]. Typically, the anatomopathological examination reveals a thickened fascia with inflammatory infiltrates that are usually perivascular and include lymphocytes [9,10] (mainly CD8+ T cells, CD4/CD8 ratio <1) [10], with a variable percentage of eosinophilic granulocytes. Eosinophils are not essential for diagnosis [9,22]. In fact, they may be absent at the late stage of the disease or shortly after the start of corticosteroid therapy [10], which is why it seems preferable to refer to the condition by the term “eosinophilic fasciitis” rather than by that of “eo-sinophilic Fasciitis”. Fasciitis to eosinophiles [19]. Inflammatory infiltrates may also include macrophages (41%) and plasma cells (44%-50%) and, more rarely, polymorphonuclear cells (6%-10%). At a more advanced stage, the fascia is less inflammatory and invaded by collagen fibrosis in almost 40% of cases [9].

Since its initial description, Shulman's syndrome has been nosologically located close to scleroderma, but the question of whether it is a simple clinical form of sclerodermia or a truly new entity has not been resolved [23,24]. Sclerodermiform states [24], characterized by chronic skin fibrosis with excessive local collagen accumulation, are the most problematic in differential diagnosis, in relation to systemic scleroderma, localized forms of sclerodermia, such as deep morphosis, and eosinophilia-droma myalgia syndrome.

Instead of the terms “sclerodermiform states” or “variants of scleroderma”, Naschitz et al. [25], in 1996, proposed to call “paniculitis fasciitis syndrome” a group of disorders with common characteristics (chronic inflammation and fibrous thickening of the subcutaneous septa, fascia and perimysium), including Shulman's phasciitis, deep morphosis, lupic paniculitis, toxic oil syndrome, myalgia-eosinophilia syndrome, transplant-to-host reaction, postirradiation reactions, lymphoedema and lipodermatosclerosis of chronic vein failure, the differentiation between these different entities is a real challenge,In morphosis, lesions consist of thickening of the dermis and varying degrees of subcutaneous tissue infiltration. However, the thickening of fascias is limited compared to what is observed in the FE [3]. Systemic sclerosis is not associated with peripheral eosinophilia or a significant corticosteroid response and is often accompanied by visceral (pulmonary or digestive) injury and capilloscopic abnormalities absent during FE [10]. Eosinophilia-myalgia syndrome has clinical and histological characteristics similar to FE, but has a history of L-tryptophan ingestion, polyneuropathy and pulmonary symptoms.

If the differential diagnosis of systemic scleroderma cannot be established, capilloscopy can provide interesting information. For eosinophilic fasciitis, it is normal or at least does not show the almost constant megacapillaries of scleroderma [26].

There is no therapeutic consensus. The basic treatment is corticosteroid therapy. With a variable initial dose, usually between 0.5 and 2 mg/kg/day, laboratory parameters quickly normalize and arthralgia and myalgia disappear. The regression of subcutaneous induration is highly variable and requires prolonged corticosteroid therapy with side effects. In a series of 52 cases, Lakhanpal et al. [27] 34 corticosteroids (initial doses of 40-60 mg/day) with 5 complete remissions, twenty partial responses and 9 failures. According to these authors, the longer or shorter duration of disease progression before introduction of corticosteroid therapy does not make any difference in response. Histologically, the inflammatory component is highly corticosensitive, while the fibrous component stabilizes or even progresses. The irregular effectiveness of corticosteroid therapy led to other treatments being tried. However, as Shulman syndrome is rare, no randomized therapeutic trials have been conducted.

Another nonconsensual point concerns the use of immunosuppressants such as azathioprine, cyclophosphamide, methotrexate, cyclosporine and more recently biotherapies (anti-TNF, rituximab) [9,10]. Their prescription is essentially justified by treatment failure or corticosteroid dependence at a high level. In the patient population studied by Lebeaux, the 32 long-term follow-up patients all received corticosteroids for an average duration of 45.7 months. Due to unsatisfactory clinical response, 44% of them (14 patients) needed second-line therapy including adjuvant immunosuppressive therapy for an average duration of 24.7 months (methotrexate in 86% of cases, azathioprine in 14% and hydroxychloroquine in 6%). 94% (17/18) of the patients treated with steroids alone achieved complete remission, compared to only 36% (5/14) of patients receiving steroids with adjuvant immunosuppressant therapy with more severe disease. These authors also show that the use of high doses of steroids as induction therapy (Solu-Medrol 0.5-1 g/day for 3 days) appears to be associated with more complete remissions [9]. On the other hand, some recent publications report the effectiveness of anti-TNF-alpha (tumor necrosis factor) (infliximab) in cases resistant to steroids and immunosuppressants [28]. FE is a disease with a good prognosis, if it is not associated with serious hematological disorders, good evolution of our patient on corticosteroid boluses and methotrexate-type immunosuppressants [29].

## Conclusion

The diagnosis of Shulman's syndrome is relatively straightforward, as it is characterized by a combination of clinical, laboratory, and histological manifestations. However, it can be challenging to consider this diagnosis due to its rarity. MRI plays a crucial role in the diagnosis, revealing characteristic fascial abnormalities and aiding in guiding the surgeon to the optimal biopsy site. Oral corticosteroids remain the mainstay of treatment and can be combined with immunosuppressive therapy, such as methotrexate, especially in patients with an unsatisfactory response to corticosteroids.

Eosinophilic fasciitis generally has a good prognosis and often resolves with treatment, except in cases with severe hematological complications. Spontaneous improvement has also been observed.

## Patient consent

For the case report written informed consent for publication of the case was obtained by the first author of the case report.
